# Diagnostic and Prognostic Value of Serum Surfactant Protein D in Interstitial Lung Disease: A Systematic Review

**DOI:** 10.7759/cureus.112026

**Published:** 2026-07-03

**Authors:** Abdulrahman Eltayeb Abdalla Abdelgadir, Doaa Amir, Khaled Abdulbasit Fadlallah Mohammed, Lima Gharbawi, Abdalla Fathi Salih, Essra Hassan Adam Alradi, Yara Ahmed

**Affiliations:** 1 Geriatric Medicine, Watford General Hospital, Watford, GBR; 2 Respiratory Medicine, Blackpool Victoria Hospital, Blackpool, GBR; 3 General Surgery, University Hospitals Coventry and Warwickshire NHS Trust, Coventry, GBR; 4 Ear, Nose, and Throat, Portsmouth Hospitals University NHS Trust, Hampshire, GBR; 5 Internal Medicine, Royal Blackburn Teaching Hospital, Blackburn, GBR; 6 Emergency Medicine, Almoosa Specialist Hospital, Al-Hasa, SAU; 7 Faculty of Medicine, University of Medical Sciences and Technology (UMST), Khartoum, SDN

**Keywords:** biomarker, diagnosis, interstitial lung disease, prognosis, pulmonary fibrosis, surfactant protein d, systematic review

## Abstract

Serum surfactant protein D (SP-D), secreted by alveolar type II pneumocytes, leaks into the circulation during alveolar epithelial injury, making it a candidate biomarker for interstitial lung disease (ILD). This systematic review appraises its contemporary diagnostic and prognostic value across ILD subtypes. PubMed, Scopus, Web of Science, and Embase were searched for original studies published from 2021 to 2025 reporting serum SP-D in adult ILD patients. Study selection, data extraction, and quality assessment (Newcastle-Ottawa Scale) followed the Preferred Reporting Items for Systematic Reviews and Meta-Analyses 2020 guidelines. Narrative synthesis was performed because of heterogeneity in populations, assay platforms, and outcomes. Ten studies enrolling 4,179 participants were included. Serum SP-D was significantly elevated in ILD versus controls in most studies, with diagnostic area under the curve values of 0.65-0.89. It correlated with forced vital capacity, diffusing capacity of the lungs for carbon monoxide, and radiological extent across phenotypes, and independently predicted progression in rheumatoid arthritis-associated ILD (hazard ratio = 1.003; p = 0.008). In acute exacerbation, SP-D uniquely correlated with coagulation markers, suggesting a distinct pathobiological role not captured by Krebs von den Lungen-6 (KL-6). Prognostic utility was limited in antifibrotic-treated idiopathic pulmonary fibrosis. KL-6 outperformed SP-D as a standalone marker, but SP-D provided complementary, non-redundant value in multi-biomarker strategies. Serum SP-D is a clinically relevant biomarker of alveolar injury in ILD, best utilised within multi-marker panels. Assay standardisation and prospective validation of SP-D-inclusive biomarker strategies remain priorities for future research.

## Introduction and background

Interstitial lung disease (ILD) encompasses a heterogeneous group of more than 200 diffuse parenchymal lung disorders characterised by varying degrees of alveolar inflammation, interstitial fibrosis, and remodelling of the pulmonary parenchyma [1]. Despite phenotypic diversity, the clinical consequence of progressive fibrosis is shared across subtypes: inexorable decline in gas exchange, exercise intolerance, and, in a substantial proportion of patients, premature death [2]. Population-based estimates indicate an annual ILD incidence of 1-31.5 cases per 100,000 individuals and a prevalence of 6.3-71 per 100,000, with idiopathic pulmonary fibrosis (IPF), connective tissue disease-associated interstitial lung disease (CTD-ILD), and hypersensitivity pneumonitis (HP) collectively accounting for the majority of cases [3]. Approximately one-third of all ILD patients develop a progressive fibrosing phenotype, and acute exacerbations (AE-ILD), defined as acute, clinically significant respiratory deteriorations of unknown cause superimposed on established ILD, carry a short-term mortality exceeding 50% [4]. While antifibrotic agents such as pirfenidone and nintedanib attenuate forced vital capacity (FVC) decline in IPF and selected progressive fibrosing ILDs, no curative therapy exists, and early, accurate disease characterisation and monitoring remain critical unmet clinical needs [5].

Current diagnostic pathways for ILD rely on a multidisciplinary synthesis of clinical history, pulmonary function testing (PFT), high-resolution computed tomography (HRCT), and, where required, histopathological sampling through transbronchial lung cryobiopsy or surgical lung biopsy [6]. Although HRCT has transformed ILD diagnosis, its radiation burden, cost, and dependence on specialist radiology expertise make it unsuitable as a frequent monitoring tool; invasive sampling is poorly tolerated in physiologically impaired patients and carries a non-negligible procedural risk [7]. These constraints have generated sustained interest in circulating serum biomarkers capable of non-invasively reflecting alveolar epithelial injury, fibrotic burden, and disease trajectory. Krebs von den Lungen-6 (KL-6), a high-molecular-weight mucin glycoprotein expressed on hyperplastic type II pneumocytes, is the most extensively validated ILD serum biomarker and is incorporated into clinical practice in several countries, particularly Japan [8]. However, KL-6 assay platforms are not universally standardised at a global level, and their performance in early-stage or non-fibrotic ILD subtypes remains variable, underscoring the need to evaluate complementary circulating markers.

Surfactant protein D (SP-D) is a collagen-containing C-type lectin of the collectin family constitutively secreted by alveolar type II pneumocytes and Clara cells into the alveolar lining fluid [9]. Under physiological conditions, SP-D serves as a first-line innate immune mediator, modulating inflammatory signalling, facilitating pathogen opsonisation, and maintaining surfactant homeostasis. In ILD, disruption of the alveolar epithelial barrier causes SP-D to leak into the systemic circulation at concentrations proportional to the degree of epithelial injury, rendering serum SP-D a molecular reflector of alveolar damage and type II pneumocyte hyperplasia, pathological processes shared by virtually all fibrotic ILD subtypes [10]. Prior investigations have demonstrated SP-D elevation in IPF, CTD-ILD, and myositis-associated ILD, with correlations reported with radiological disease extent, pulmonary function, and clinical outcomes [11]. Importantly, SP-D and KL-6 are not biologically interchangeable: whereas KL-6 is released chiefly from hyperplastic and regenerating type II pneumocytes and thus indexes fibroproliferative epithelial activity, SP-D reflects the integrity and functional state of the alveolar surfactant system and the acuity of epithelial injury. The two markers may therefore capture complementary, non-identical dimensions of ILD pathobiology, providing the rationale for the direct comparison undertaken here. Nevertheless, the published literature has been characterised by substantial heterogeneity in study populations, assay platforms, and outcome measures, preventing clear evidence-based guidance on SP-D’s clinical utility.

To date, no systematic review has comprehensively and exclusively appraised the contemporary evidence on the diagnostic and prognostic value of serum SP-D across the full spectrum of ILD subtypes using literature published from 2021 onwards, a period marked by the emergence of large prospective cohort data, population-level fibrosis screening programmes, and novel characterisation of SP-D in AE-ILD and antifibrotic treatment contexts. The present systematic review was therefore undertaken with four pre-specified objectives: (i) to evaluate the diagnostic accuracy of serum SP-D for ILD detection and severity classification; (ii) to appraise the prognostic value of SP-D for disease progression, AE-ILD, and mortality; (iii) to examine correlations of SP-D with pulmonary function and radiological disease extent; and (iv) to contextualise SP-D performance relative to established comparator biomarkers, most notably KL-6 and matrix metalloproteinase-7 (MMP-7). A central translational motivation for this appraisal is to clarify where, if anywhere, a low-cost, blood-based marker such as SP-D could be implemented in routine ILD care, for non-invasive disease monitoring, risk stratification, or as a complement to KL-6, given the radiation burden, cost, and limited accessibility of repeated HRCT and the invasiveness of histopathological sampling. The potential advantage of SP-D over established markers lies not in superior standalone accuracy but in its distinct biological signal of acute alveolar epithelial injury, which may add non-redundant information when interpreted alongside KL-6; the clinical and economic value of such a combination is examined throughout this review.

## Review

Methodology

Study Design

This systematic review was conducted and reported in accordance with the Preferred Reporting Items for Systematic Reviews and Meta-Analyses (PRISMA) 2020 statement [12]. The research question was framed according to the Population, Index test, Comparator, and Outcome (PICO) framework: the population comprised adult patients (aged ≥18 years) with any confirmed ILD subtype; the index test was the measurement of serum SP-D; the comparators included non-ILD disease controls, healthy individuals, and alternative serum biomarkers; and the outcomes encompassed ILD diagnosis, disease progression, AE-ILD, mortality, and correlation with pulmonary function and radiological parameters.

Protocol and Registration

The objectives, eligibility criteria, search strategy, and synthesis approach were pre-specified before screening and are reported in full below to support transparency and reproducibility. A formal protocol was not prospectively registered in PROSPERO or an equivalent registry; this is acknowledged as a limitation. The review was otherwise conducted and reported in accordance with the PRISMA 2020 statement [12].

Eligibility Criteria

Eligibility criteria were established using the PICOS framework (Table 1). Studies meeting all inclusion criteria and none of the exclusion criteria were considered eligible for inclusion in this review. Case reports and small case series (fewer than 10 participants) were excluded to limit the influence of unstable point estimates and reporting bias inherent to very small samples and to preserve the methodological comparability of included cohorts; the potential consequence of this decision for under-represented or rare ILD subtypes is addressed in the Limitations.

**Table 1:** PICOS framework and eligibility criteria. ILD = interstitial lung disease; SP-D = surfactant protein D; KL-6 = Krebs von den Lungen-6; MMP-7 = matrix metalloproteinase-7

PICOS element	Inclusion criteria	Exclusion criteria
Population (P)	Adults (≥18 years) diagnosed with any ILD subtype according to established international guidelines	Paediatric populations; animal studies; in vitro studies; studies where ILD was not the primary clinical focus
Index Test/Exposure (I)	Serum SP-D measured as a biomarker, exposure variable, or prognostic factor	Studies measuring SP-D exclusively in bronchoalveolar lavage fluid without corresponding serum measurements
Comparator (C)	Healthy controls, non-ILD controls, alternative biomarkers (e.g., KL-6, SP-A, MMP-7), or internal disease severity/progression groups	No relevant comparator or reference group where applicable
Outcomes (O)	Diagnostic accuracy, disease progression, mortality, acute exacerbation, pulmonary function parameters, radiological extent, or biomarker correlations	No quantitative SP-D-related clinical outcomes reported
Study Design (S)	Original peer-reviewed observational studies (prospective, retrospective, cross-sectional, longitudinal, cohort) published in English between 1 January 2021 and 31 December 2025	Reviews, systematic reviews, meta-analyses, editorials, letters, conference abstracts, case reports, case series (<10 participants), preprints not subsequently published in peer-reviewed form

Information Sources and Search Strategy

A systematic literature search was conducted in PubMed/MEDLINE, Scopus, Web of Science, and Embase covering the period from 1 January 2021 to 31 December 2025. The database searches were last executed on 31 December 2025, which constitutes the final search date for this review; no further records were added thereafter. The publication window was restricted to 2021-2025 for the following three reasons: (i) to capture the contemporary evidence base that emerged after the 2018 American Thoracic Society/European Respiratory Society/Japanese Respiratory Society/Latin American Thoracic Society IPF guideline and the regulatory expansion of antifibrotic therapy to progressive fibrosing ILDs, a period in which serum biomarker evaluation in ILD intensified; (ii) to reflect SP-D measurement using current-generation, commercially standardised immunoassays rather than heterogeneous legacy in-house assays; and (iii) to focus the synthesis on novel contexts, large prospective cohorts, population-level fibrosis screening, and SP-D characterisation in acute exacerbation and antifibrotic-treatment settings, that distinguish recent literature from earlier foundational work. The search was independently performed by two reviewers. Reference lists of all included studies were additionally screened to identify potentially eligible articles not captured through database searching. The complete search strategy for each database is presented in Table 2.

**Table 2:** Complete database search strategy.

Database	Search string
PubMed/MEDLINE	(“Surfactant Protein D”[Mesh] OR “surfactant protein D” OR “SP-D” OR “surfactant protein-D” OR “pulmonary collectin D”) AND (“Interstitial Lung Diseases”[Mesh] OR “interstitial lung disease” OR ILD OR “pulmonary fibrosis” OR “idiopathic pulmonary fibrosis” OR IPF OR “connective tissue disease-associated interstitial lung disease” OR CTD-ILD OR “hypersensitivity pneumonitis” OR “interstitial pneumonia” OR “progressive fibrosing interstitial lung disease” OR “acute exacerbation of interstitial lung disease”) Filters: Humans, English, 2021–2025
Scopus	TITLE-ABS-KEY (“surfactant protein D” OR “SP-D” OR “surfactant protein-D” OR “pulmonary collectin D”) AND TITLE-ABS-KEY (“interstitial lung disease” OR ILD OR “pulmonary fibrosis” OR “idiopathic pulmonary fibrosis” OR IPF OR “connective tissue disease-associated interstitial lung disease” OR CTD-ILD OR “hypersensitivity pneumonitis” OR “interstitial pneumonia” OR “progressive fibrosing interstitial lung disease” OR “acute exacerbation of interstitial lung disease”) AND PUBYEAR > 2020 AND PUBYEAR < 2026
Web of Science	TS=(“surfactant protein D” OR “SP-D” OR “surfactant protein-D” OR “pulmonary collectin D”) AND TS=(“interstitial lung disease” OR ILD OR “pulmonary fibrosis” OR “idiopathic pulmonary fibrosis” OR IPF OR “connective tissue disease-associated interstitial lung disease” OR CTD-ILD OR “hypersensitivity pneumonitis” OR “interstitial pneumonia” OR “progressive fibrosing interstitial lung disease” OR “acute exacerbation of interstitial lung disease”), refined by English language and publication years 2021–2025
Embase	(‘surfactant protein d’/exp OR ‘surfactant protein d’ OR ‘sp-d’ OR ‘pulmonary collectin d’) AND (‘interstitial lung disease’/exp OR ‘interstitial lung disease’ OR ild OR ‘pulmonary fibrosis’ OR ‘idiopathic pulmonary fibrosis’ OR ipf OR ‘connective tissue disease-associated interstitial lung disease’ OR ‘ctd-ild’ OR ‘hypersensitivity pneumonitis’ OR ‘interstitial pneumonia’ OR ‘progressive fibrosing interstitial lung disease’ OR ‘acute exacerbation of interstitial lung disease’) AND [humans]/lim AND [english]/lim AND (2021-2025)/py

Study Selection

Title and abstract screening was conducted independently by two reviewers against the pre-specified eligibility criteria. Discrepancies were resolved by discussion and consensus, with a third reviewer serving as arbitrator where agreement could not be reached. Records identified across the four databases were de-duplicated before screening, and full-text retrieval was attempted for all potentially eligible records.

Data Extraction

Structured data extraction was performed independently by the same two reviewers using a pre-piloted standardised extraction form. The following information was collected from each included study: first author, year of publication, country of origin, study design (prospective or retrospective; longitudinal or cross-sectional; single- or multicentre), ILD subtype(s) and the diagnostic criteria applied, total sample size, participant demographic characteristics (mean age, sex distribution), SP-D assay methodology (immunoassay platform, kit manufacturer, reported cut-off value), co-measured biomarkers, nature of the comparator or control group, primary and secondary outcome definitions, statistical methods employed, and all quantitative SP-D-related findings, including diagnostic accuracy metrics (sensitivity, specificity, area under the curve (AUC)), correlation coefficients with PFT parameters, and effect estimates for progression or mortality (hazard ratios (HRs), odds ratios (ORs)). Any discrepancies in extracted data were resolved through re-examination of the source article and discussion between the two reviewers, with adjudication by the third reviewer where required.

Quality Assessment

The methodological quality of all included studies was independently assessed by two reviewers using the Newcastle-Ottawa Scale (NOS) [13], which evaluates observational studies across the following three domains: selection of study groups (maximum 4 stars), comparability of groups (maximum 2 stars), and ascertainment of exposure or outcome (maximum 3 stars), yielding a maximum total score of 9 stars. Studies scoring 8-9 stars were classified as high quality, those scoring 6-7 stars as moderate quality, and those scoring 5 or fewer stars as low quality. Scoring discrepancies between the two reviewers were resolved by discussion and consensus, with the third reviewer adjudicating any unresolved disagreement. Quality assessment scores were not used as grounds for study exclusion but were incorporated into the interpretation of findings and the weighting of conclusions.

Data Synthesis and Rationale for the Narrative Approach

A formal meta-analysis was considered during the protocol phase but was ultimately determined to be methodologically unjustifiable for three substantive reasons. First, there was profound clinical and population heterogeneity across included studies: ILD subtypes ranged from rheumatoid arthritis-associated interstitial lung disease (RA-ILD) and interstitial pneumonia with autoimmune features (IPAF) to IPF, chronic fibrosing interstitial pneumonia, mixed ILD populations, and AE-ILD, with no two studies enrolling clinically equivalent patient populations. Pooling SP-D estimates across these phenotypically distinct groups would violate the fundamental meta-analytic assumption of a common underlying effect size and would produce a composite estimate that is biologically uninterpretable and clinically misleading. Second, there was substantial methodological heterogeneity in SP-D measurement: while the majority of studies employed the Yamasa Corporation enzyme-linked immunosorbent assay (ELISA) at the established 110 ng/mL threshold, others used latex-enhanced immunoturbidimetric assays, chemiluminescent enzyme immunoassays, or manufacturer-specific references without reporting cut-off values, generating systematic between-study measurement variance that cannot be corrected by standard statistical pooling techniques. Third, the reported outcome metrics were fundamentally non-commensurable across studies, encompassing diagnostic AUC values with sensitivity and specificity pairs, Pearson and Spearman correlation coefficients with individual PFT parameters, Cox regression HRs for progression or mortality, and unstandardised mean differences in SP-D concentrations between clinical subgroups, and cannot be mathematically aggregated without distorting the clinical information they convey. In this context, a narrative synthesis was adopted as the most methodologically rigorous and clinically informative approach. Where available, key quantitative estimates from each study are summarised to facilitate cross-study comparison. Because no quantitative pooling was undertaken, statistical small-study/publication-bias diagnostics that require a meta-analytic dataset, such as funnel-plot asymmetry inspection and Egger’s regression test, could not be meaningfully applied; the potential for publication and small-study bias is instead addressed qualitatively in the Limitations. For the same reason, a formal GRADE (Grading of Recommendations Assessment, Development, and Evaluation) assessment of the certainty of evidence, which is calibrated to pooled effect estimates from comparable outcomes, was not performed; the strength of the evidence is instead appraised descriptively in the Discussion and Limitations, taking into account study design, sample size, directness, and consistency across the included studies.

Results

Study Selection

**Figure 1:**
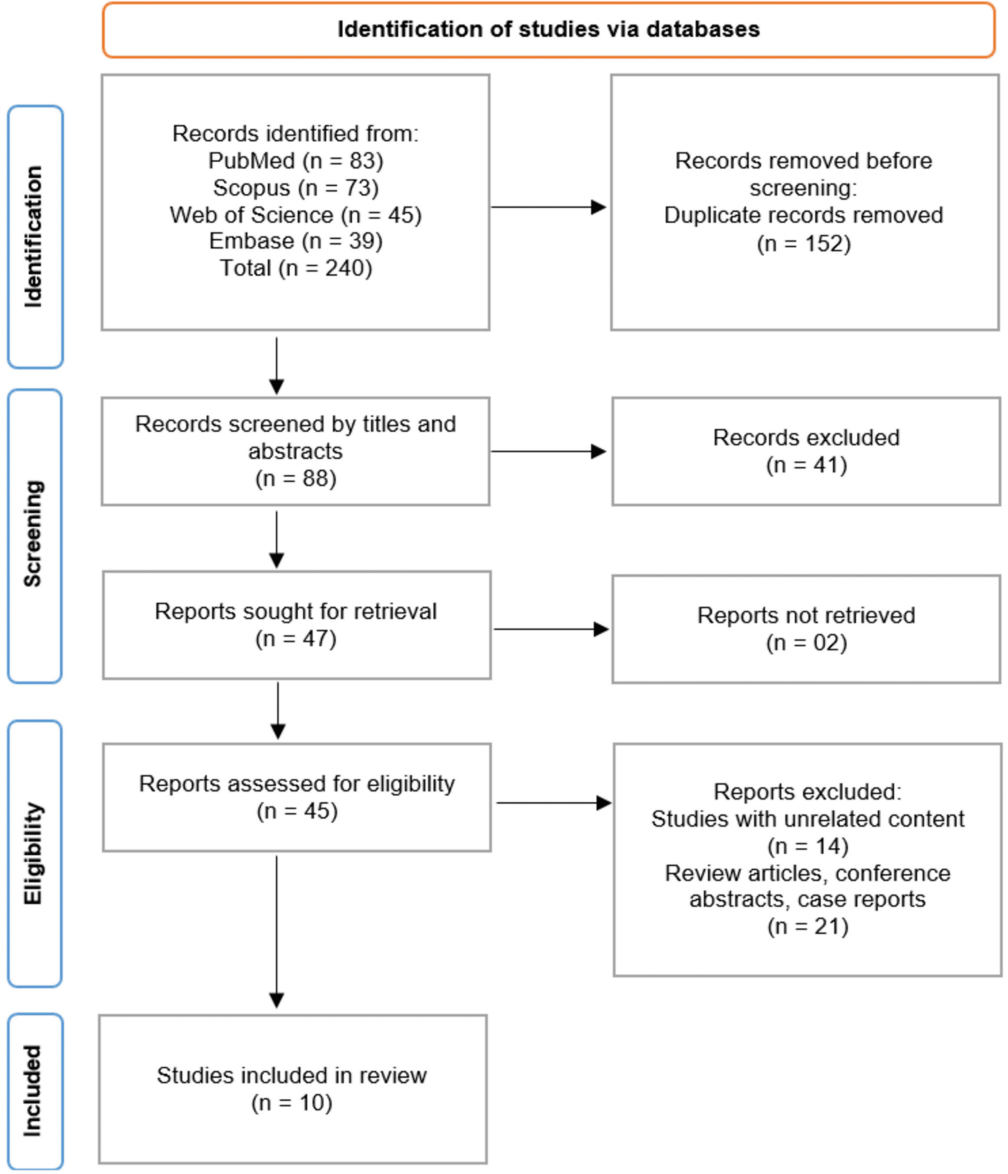
Preferred Reporting Items for Systematic Reviews and Meta-Analyses (PRISMA) flowchart.

The systematic database search retrieved 240 records in total: 83 from PubMed, 73 from Scopus, 45 from Web of Science, and 39 from Embase. After automated and manual removal of 152 duplicate records, 88 unique records remained for title and abstract screening. Following independent dual-reviewer screening, 41 records were excluded for irrelevance, and the remaining 47 were sought for full-text retrieval. Two reports could not be retrieved, leaving 45 reports for full-text eligibility assessment. Of these, 35 were excluded: 14 for content unrelated to the research question, and 21 because they were review articles, conference abstracts, or case reports rather than original research. Ten studies [14-23] met all eligibility criteria and were included in the final synthesis. The full selection process is summarised in the PRISMA 2020 flow diagram (Figure 1).

Overview of Included Studies

The 10 included studies [14-23] were published between 2021 and 2025 and collectively enrolled 4,179 participants across six countries: Japan (n = 3), South Korea (n = 2), Poland (n = 2), the United States (n = 1), India (n = 1), and China (n = 1). Individual sample sizes ranged from 28 to 3,022 participants. Six studies were prospective [14-16,21-23] and four were retrospective [17-20]. The ILD populations studied were heterogeneous, encompassing RA-ILD [16,22], IPF [19,23], CTD-ILD and IPAF [15,17], AE-ILD [18], mixed ILD populations [20,21], and a general health-checkup population screened for suspected lung fibrosis [14]. Serum SP-D was most frequently co-measured with KL-6 (nine studies), MMP-7 (four studies), and SP-A (four studies). Full characteristics of included studies are presented in Table 3.

**Table 3:** Characteristics of included studies. ILD = interstitial lung disease; HRCT = high-resolution computed tomography; IPAF = interstitial pneumonia with autoimmune features; CTD-ILD = connective tissue disease-associated interstitial lung disease; RA-ILD = rheumatoid arthritis-associated interstitial lung disease; CHP = chronic hypersensitivity pneumonitis; iNSIP = idiopathic non-specific interstitial pneumonia; AE-ILD = acute exacerbations of interstitial lung disease; IPF = idiopathic pulmonary fibrosis; HP = hypersensitivity pneumonitis

Study (author, year)	Country	Study design	ILD subtype/population	N	Mean age (years)	Female (%)
Nishikiori et al. [14] (2025)	Japan	Prospective observational (health checkup cohort)	Mixed ILD subtypes suspected on HRCT (general screening population)	3,022	64.0 ± 12.5	42.3%
Miądlikowska et al. [15] (2025)	Poland	Prospective observational (one-year follow-up)	IPAF (n = 24), CTD-ILD (n = 21), CTD without ILD (n = 23)	68	59.4 ± 12.1	78.0%
Chang et al. [16] (2025)	South Korea/USA	Prospective multicentre cohort (KORAIL; median three-year follow-up)	RA-ILD confirmed by chest CT	136	66.4 ± 9.5	69.6%
Maharjan et al. [17] (2024)	USA	Retrospective cross-sectional (single centre)	CTD-ILD (n = 25), other ILD (n = 39), non-ILD (n = 58), healthy controls (n = 120)	242	57.2 ± 14.3	71.1%
Takeshita et al. [18] (2024)	Japan	Retrospective cohort (single centre)	Stable ILD (IPF, CTD-ILD, CHP, iNSIP) vs. AE-ILD	81	72.1 ± 9.8	33.3%
Wakamatsu et al. [19] (2024)	Japan	Retrospective (single centre; 2008–2021)	Chronic fibrosing IP (IPF, iNSIP)	39	70.3 ± 8.5	38.5%
Wang et al. [20] (2024)	China	Retrospective cross-sectional (single centre)	Mixed ILD (IPF, CTD-ILD, HP, other) vs. non-ILD controls	366	58.6 ± 13.2	48.8%
Rai et al. [21] (2023)	India	Prospective observational (six-month longitudinal)	Mixed ILD (IPF, CTD-ILD, HP, sarcoidosis)	44	52.3 ± 14.1	38.6%
Moon et al. [22] (2021)	South Korea	Prospective multicentre cohort (KORAIL baseline)	RA-ILD confirmed by chest CT	153	63.2 ± 9.6	71.9%
Majewski et al. [23] (2021)	Poland	Prospective exploratory cohort (≤24-month serial measurements)	IPF on antifibrotic therapy (pirfenidone or nintedanib)	28	68.4 ± 7.5	17.9%

Serum Surfactant Protein D Levels in Interstitial Lung Disease Versus Controls

Eight studies compared serum SP-D concentrations in ILD patients against non-ILD or healthy control populations. In the majority, SP-D was significantly elevated in ILD. Maharjan et al. [17] demonstrated markedly elevated SP-D in ILD versus non-ILD patients (p < 0.0001), and Miądlikowska et al. [15] confirmed that SP-D was specifically elevated in IPAF and CTD-ILD compared with CTD patients without lung involvement (p = 0.0005), indicating that SP-D elevation reflects pulmonary parenchymal injury rather than systemic autoimmune inflammation per se. In the prospective health checkup screening cohort of Nishikiori et al. [14], serum SP-D at the 110 ng/mL threshold identified individuals with HRCT-confirmed lung fibrosis with an AUC of 0.79. In contrast, Wang et al. [20] reported a discordant finding: SP-D levels did not differ significantly between ILD and non-ILD controls (p > 0.05) in a heterogeneous Chinese cohort, possibly reflecting a predominantly early-stage or non-fibrotic ILD distribution, assay-platform differences, or ethnic variation in SP-D reference ranges.

Diagnostic Performance of Serum Surfactant Protein D

Four studies formally evaluated the diagnostic accuracy of serum SP-D using receiver operative characteristics curve analysis [14,17,18,20], with AUC values ranging from 0.65 to 0.89. Maharjan et al. [17] reported the highest diagnostic accuracy, i.e., sensitivity of 77%, specificity of 93%, and AUC of 0.89, in a CTD-ILD-enriched cohort, and found that the combination of SP-D with KL-6 yielded accuracy comparable to the full three-biomarker panel, supporting an efficient two-marker diagnostic strategy. Nishikiori et al. [14] achieved an AUC of 0.79 for SP-D alone in a general screening population, improving to 0.83 when SP-D and KL-6 were integrated with a deep-learning chest radiograph algorithm. Takeshita et al. [18] reported an AUC of 0.78 for SP-D in discriminating AE-ILD from stable ILD, accompanied by significantly higher SP-D concentrations in AE-ILD (median = 251 vs. 122 ng/mL, p < 0.001). A distinctive observation from this study was that SP-D, but not KL-6, correlated significantly with coagulation and fibrinolysis markers, including thrombin-antithrombin III complex (TAT), D-dimer, and plasmin-α2-plasmin inhibitor complex (PIC) in AE-ILD (r = 0.52-0.68), suggesting that SP-D captures a pathobiological dimension of acute alveolar epithelial injury coupled with secondary coagulopathy that is not reflected by KL-6. Wang et al. [20] found SP-D diagnostically inferior to KL-6 and MMP-7 (AUC = 0.65), confirming that SP-D diagnostic performance is context- and population-dependent.

Correlation of Serum Surfactant Protein D with Pulmonary Function and Radiological Extent

Seven studies examined associations between serum SP-D and PFT parameters, principally FVC and diffusing capacity of the lungs for carbon monoxide (DLCO) [14-16,20-23]. Moon et al. [22] demonstrated in 153 RA-ILD patients that SP-D was significantly and negatively correlated with FVC (r = −0.250, p = 0.002) and DLCO (r = −0.286, p = 0.001), and increased progressively across semiquantitative CT lung involvement grades (p < 0.001). Miądlikowska et al. [15] confirmed correlations of SP-D with HRCT extent of ILD (r = 0.35, p = 0.02), six-minute walk distance, and oxygen saturation in IPAF. Rai et al. [21] provided longitudinal evidence that serial changes in SP-D over six months tracked corresponding changes in FVC (p = 0.008), DLCO (p = 0.012), forced expiratory volume in one second (p = 0.046), and arterial PaO_2_ (p = 0.020), supporting the utility of serial SP-D measurement as a functional monitoring biomarker in mixed ILD. In all studies reporting functional correlations, MMP-7 demonstrated stronger associations with CT lung involvement than SP-D, while KL-6 showed more consistent correlations with DLCO (Table 4).

**Table 4:** SP-D assay characteristics, co-measured biomarkers, comparator groups, primary outcomes, and key SP-D findings. SP-D = surfactant protein D; ELISA = enzyme-linked immunosorbent assay; HRCT = high-resolution computed tomography; KL-6 = Krebs von den Lungen-6; AI = artificial intelligence; AUC = area under the curve; TGF-β1 = transforming growth factor-beta 1; ILD = interstitial lung disease; CTD-ILD = connective tissue disease-associated interstitial lung disease; FVC = forced vital capacity; TLCO = transfer factor of the lung for carbon monoxide; 6MWD = six-minute walk distance; IPAF = interstitial pneumonia with autoimmune features; MMP-7 = matrix metalloproteinase-7; RA-ILD = rheumatoid arthritis-associated interstitial lung disease; PPF = progressive pulmonary fibrosis; ATS = American Thoracic Society; ERS = European Respiratory Society; TAT = thrombin-antithrombin III complex; PIC = plasmin-α2-plasmin inhibitor complex; BALF = bronchoalveolar lavage fluid; CFIP = chronic fibrosing interstitial pneumonia; DLCO = diffusing capacity of the lungs for carbon monoxide; FEV1 = forced expiratory volume in one second; CA-19-9 = carbohydrate antigen 19-9; CA-125 = cancer antigen 125; CCL18 = C-C motif chemokine ligand 18; HR = hazard ratio; CI = confidence interval

Study (author, year)	SP-D assay method and cut-off	Co-measured biomarkers	Comparator/Control group	Primary outcome(s)	Key SP-D findings
Nishikiori et al. [14] (2025)	ELISA (Yamasa Corp.); cut-off 110 ng/mL	KL-6; deep learning chest radiograph AI	Subjects without lung fibrosis on HRCT	Diagnostic accuracy of SP-D and KL-6 for HRCT-confirmed lung fibrosis	SP-D AUC 0.79 for HRCT-confirmed fibrosis; combining SP-D + KL-6 + AI improved early detection (AUC = 0.83)
Miądlikowska et al. [15] (2025)	ELISA (Yamasa Corp.); cut-off not specified	KL-6; TGF-β1	CTD patients without ILD (n = 23)	Correlation of SP-D with lung function (FVC, TLCO, 6MWD, SpO_2_), HRCT extent, and one-year ILD progression	SP-D significantly higher in IPAF/CTD-ILD vs. CTD without ILD (p = 0.0005); correlated with HRCT extent (r = 0.35, p = 0.02), 6MWD and SpO_2_; no independent prediction of short-term progression
Chang et al. [16] (2025)	ELISA; manufacturer reference range	KL-6; MMP-7	Stable RA-ILD (internal comparison by PPF progression status)	Risk of RA-ILD progression by PPF criteria (ATS/ERS 2022); multivariable Cox regression	Baseline SP-D independently associated with progression (HR = 1.003, 95% CI = 1.001–1.006; p = 0.008); serial change in KL-6 a stronger predictor than change in SP-D
Maharjan et al. [17] (2024)	ELISA (Radiometer); specific cut-off not reported	KL-6; SP-A	Healthy controls (n = 120) and non-ILD patients (n = 58)	Diagnostic accuracy (AUC, sensitivity, specificity) of SP-D, KL-6, and SP-A for ILD diagnosis and severity classification	SP-D sensitivity 77%, specificity 93%, AUC 0.89; SP-D + KL-6 yielded accuracy comparable to full panel; no significant SP-D difference between CTD-ILD and other ILD subtypes (p ≥ 0.05)
Takeshita et al. [18] (2024)	ELISA (latex agglutination); cut-off 110 ng/mL	KL-6; TAT; D-dimer; PIC	Stable ILD outpatients (n = 63)	Ability of SP-D and coagulation markers to distinguish AE-ILD from stable ILD; correlation among markers in AE-ILD	SP-D significantly elevated in AE-ILD vs stable ILD (median = 251 vs. 122 ng/mL; AUC = 0.78); SP-D positively correlated with D-dimer and PIC (r = 0.52–0.68); KL-6 showed no significant correlation with coagulation markers
Wakamatsu et al. [19] (2024)	ELISA (Yamasa Corp.); serum and BALF measured; cut-off 110 ng/mL	KL-6; SP-A (serum and BALF)	Stable CFIP vs progressive CFIP (FVC decline ≥10%/year)	Prognostic value of serum and BALF SP-D, KL-6, and SP-A for CFIP progression and mortality	Serum SP-D was non-significantly elevated in progressive vs. stable CFIP; SP-D was not predictive; KL-6 and SP-A showed stronger prognostic association; SP-D was not retained on multivariate analysis
Wang et al. [20] (2024)	Chemiluminescent enzyme immunoassay (Fujirebio); ELISA; cut-off not specified	KL-6; SP-A; MMP-7	Non-ILD disease controls (n = 112)	Diagnostic accuracy of SP-D, KL-6, SP-A, MMP-7 for ILD; correlation with HRCT fibrosis score	SP-D was not significantly different between ILD and non-ILD groups (p > 0.05); KL-6 and MMP-7 were superior (AUC); SP-D AUC 0.65; SP-D showed a positive correlation with fibrosis score
Rai et al. [21] (2023)	ELISA (Yamasa Corp.); cut-off 110 ng/mL	KL-6	Internal comparison by six-month outcome (died, deteriorated, stable/improved)	Prognostic value of serum SP-D and KL-6 for functional decline and mortality over six months	SP-D was highest in patients who died (256.1 ng/mL) vs. deteriorated (191.2 ng/mL); serial change in SP-D correlated with FVC (p = 0.008), DLCO (p = 0.012), FEV1 (p = 0.046), PaO_2_ (p = 0.020); SP-D independently predicted mortality (GLM)
Moon et al. [22] (2021)	ELISA (Meso Scale Discovery); reference range per manufacturer	KL-6; MMP-7	Internal comparison across semiquantitative CT lung involvement grades (1–4)	Correlation of serum SP-D, MMP-7, and KL-6 with FVC, DLCO, and CT lung involvement grade in RA-ILD	SP-D negatively correlated with FVC (r = −0.250, p = 0.002) and DLCO (r = −0.286, p = 0.001); SP-D increased with CT grade (p < 0.001); MMP-7 showed stronger correlation with CT involvement than SP-D
Majewski et al. [23] (2021)	ELISA (Yamasa Corp.); cut-off 110 ng/mL; serial measurements at 0, 6, 12, 18, 24 months	KL-6; MMP-7; CA19-9; CA-125; CCL18; periostin	Within-patient baseline vs follow-up; stable vs progressive IPF subgroups	Serial changes in SP-D and six other biomarkers over 24 months of antifibrotic therapy; correlation with disease progression	No significant SP-D trend during antifibrotic therapy; SP-D did not distinguish progressors from stable patients; high inter-patient variability; KL-6 showed a more consistent longitudinal pattern than SP-D

Prognostic Value of Serum Surfactant Protein D

Five studies assessed the prognostic utility of serum SP-D for disease progression or mortality [15,16,19,21,23]. Rai et al. [21] provided the most direct and robust prognostic evidence: baseline SP-D was highest in patients who died within six months (mean = 256.1 ng/mL) compared with those who deteriorated (191.2 ng/mL) or remained stable, and serial changes in SP-D independently predicted mortality alongside KL-6. Chang et al. [16] demonstrated within the prospective KORAIL cohort (n = 136, median follow-up = 3 years) that baseline SP-D was an independent predictor of RA-ILD progression meeting progressive pulmonary fibrosis (PPF) criteria (HR = 1.003, 95% confidence interval = 1.001-1.006; p = 0.008) in multivariable Cox regression; however, serial change in KL-6 was the stronger predictor of progression compared with change in SP-D. In contrast, Wakamatsu et al. [19] found that serum SP-D was not significantly elevated in progressive versus stable chronic fibrosing interstitial pneumonia and was not retained as an independent predictor on multivariate analysis, with KL-6 and SP-A outperforming SP-D. Majewski et al. [23] observed no consistent SP-D trend during antifibrotic therapy in 28 IPF patients over 24 months, with high inter-patient variability and no capacity to distinguish progressors from stable patients. Miądlikowska et al. [15] similarly found that baseline SP-D did not independently predict short-term ILD progression in IPAF or CTD-ILD at one year.

Comparative Performance of Surfactant Protein D Versus Other Biomarkers

Across the nine studies that directly compared SP-D with at least one other biomarker, KL-6 demonstrated superior or equivalent performance in most diagnostic and prognostic contexts [17,19,20,22,23]. MMP-7 showed stronger correlations with CT lung involvement in RA-ILD than SP-D [22]. However, SP-D provided complementary and non-redundant value in several specific clinical settings: it correlated with coagulation markers during AE-ILD, whereas KL-6 did not [18]; it contributed incrementally to population-level fibrosis screening when combined with KL-6 and artificial intelligence (AI)-enhanced imaging [14]; it independently predicted RA-ILD progression after adjustment for KL-6 [16]; and it predicted mortality with effect sizes comparable to KL-6 in mixed ILD [21]. These observations collectively support the position that SP-D serves as a complementary rather than interchangeable member of multi-biomarker ILD panels.

Risk of Bias Assessment

The risk of bias was assessed for all 10 included studies using NOS, with scores ranging from 6 to 9 out of 9 (Table 5). Four studies were rated as high quality (NOS ≥8): Nishikiori et al. [14], Chang et al. [16], Rai et al. [21], and Moon et al. [22]. The remaining six studies were rated as moderate quality (NOS 6-7): Miądlikowska et al. [15], Maharjan et al. [17], Takeshita et al. [18], Wakamatsu et al. [19], Wang et al. [20], and Majewski et al. [23]. The most common methodological concerns were small sample sizes [15,18,19,23], retrospective single-centre designs with potential selection bias [17-20], heterogeneity in ILD phenotype composition limiting between-study comparability, and inter-study variability in SP-D assay platforms and reported cut-off thresholds. No study was rated as low quality, and no study was excluded on quality grounds.

**Table 5:** Risk of bias assessment using the Newcastle-Ottawa Scale. NOS key: ★ = one awarded star. Domains — Selection (max 4 ★): representativeness of exposed cohort, selection of non-exposed cohort, ascertainment of exposure, outcome not present at start of study; Comparability (max 2 ★): comparability of cohorts based on design or analysis; Outcome (max 3 ★): assessment of outcome, adequacy of follow-up length, adequacy of follow-up completeness. Quality classification: High = NOS 8–9; Moderate = NOS 6–7; Low = NOS ≤5. NOS = Newcastle-Ottawa Scale; HRCT = high-resolution computed tomography; ATS = American Thoracic Society; ERS = European Respiratory Society ILD = interstitial lung disease; PPF = progressive pulmonary fibrosis; SP-D = surfactant protein D; ELISA = enzyme-linked immunosorbent assay; AE-ILD = acute exacerbations of interstitial lung disease; BAL = bronchoalveolar lavage; FVC = forced vital capacity; DLCO = diffusing capacity of the lungs for carbon monoxide; IPF = idiopathic pulmonary fibrosis

Study (author, year)	Selection (maximum 4 ★)	Comparability (maximum 2 ★)	Outcome/Exposure (maximum 3 ★)	NOS score (/9)	Quality	Key risk of bias concerns
Nishikiori et al. [14] (2025)	★★★★	★★	★★★	9/9	High	Prospective; large population-based sample; validated HRCT reference standard; health-checkup cohort limits generalisability to symptomatic ILD
Miądlikowska et al. [15] (2025)	★★★	★★	★★	7/9	Moderate	Small sample (n = 68); short follow-up (one year); adequate control group; subjective ILD progression definition; single centre
Chang et al. [16] (2025)	★★★★	★★	★★★	9/9	High	Prospective multicentre (KORAIL); standardised PPF criteria (ATS/ERS 2022); adequate follow-up (median = 3 years); some missing SP-D values at follow-up
Maharjan et al. [17] (2024)	★★★	★★	★★	7/9	Moderate	Retrospective; single centre; potential referral bias; consecutive patient selection; validated ELISA assay; no prospective outcome follow-up
Takeshita et al. [18] (2024)	★★★	★★	★★	7/9	Moderate	Retrospective; small AE-ILD group (n = 18); single centre; coagulopathy was a secondary focus; clinically appropriate stable ILD comparator
Wakamatsu et al. [19] (2024)	★★★	★	★★	6/9	Moderate	Retrospective; only BAL-tested patients included (n = 39 of 173) introducing selection bias; small sample; long inclusion period (2008–2021); single centre
Wang et al. [20] (2024)	★★★	★★	★★	7/9	Moderate	Retrospective; single centre; heterogeneous ILD subtypes; non-ILD controls may not represent true clinical comparators; SP-D assay not standardised
Rai et al. [21] (2023)	★★★	★★	★★★	8/9	High	Prospective longitudinal; small sample (n = 44); mixed ILD subtypes; short follow-up (six months); validated functional outcomes (FVC, DLCO, PaO_2_)
Moon et al. [22] (2021)	★★★★	★★	★★★	9/9	High	Prospective multicentre (KORAIL); large cohort; standardised CT grading; cross-sectional baseline design limits causal inference; SP-D was not the primary study biomarker
Majewski et al. [23] (2021)	★★★	★★	★★	7/9	Moderate	Small exploratory cohort (n = 28); single centre; IPF only; antifibrotic therapy may confound SP-D levels; within-patient serial design partially mitigates selection bias

Summary of Key Diagnostic and Prognostic Estimates

To facilitate cross-study comparison in the absence of a quantitative meta-analysis, the principal diagnostic, correlative, and prognostic effect estimates for serum SP-D are summarised in Table 6.

**Table 6:** Summary of key diagnostic, correlative, and prognostic estimates for serum SP-D across included studies. SP-D = surfactant protein D; AUC = area under the curve; HRCT = high-resolution computed tomography; KL-6 = Krebs von den Lungen-6; AI = artificial intelligence; CTD-ILD = connective tissue disease-associated interstitial lung disease; AE-ILD = acute exacerbations of interstitial lung disease; TAT = thrombin-antithrombin III complex; PIC = plasmin-α2-plasmin inhibitor complex; ILD = interstitial lung disease; MMP-7 = matrix metalloproteinase-7; FVC = forced vital capacity; DLCO = diffusing capacity of the lungs for carbon monoxide; IPAF = interstitial pneumonia with autoimmune features; RA-ILD = rheumatoid arthritis-associated interstitial lung disease; PPF = progressive pulmonary fibrosis; HR = hazard ratio; CI = confidence interval

Study	Domain	Key SP-D Estimate	Comparator Note
Nishikiori et al. [14]	Diagnostic	AUC = 0.79 (SP-D alone, 110 ng/mL) for HRCT-confirmed fibrosis; 0.83 with KL-6 + AI	SP-D additive to KL-6 + AI in screening
Maharjan et al. [17]	Diagnostic	AUC = 0.89; sensitivity = 77%, specificity 93% (CTD-ILD-enriched)	SP-D + KL-6 ≈ full three-marker panel
Takeshita et al. [18]	Diagnostic (AE-ILD)	AUC = 0.78; SP-D = 251 vs. 122 ng/mL (AE vs. stable); r = 0.52–0.68 with TAT/D-dimer/PIC	SP-D, not KL-6, tracks coagulopathy
Wang et al. [20]	Diagnostic	AUC = 0.65; no significant ILD vs non-ILD difference (p > 0.05)	Inferior to KL-6 and MMP-7
Moon et al. [22]	Correlation	FVC r = −0.250 (p = 0.002); DLCO r = −0.286 (p = 0.001); rises with CT grade (p < 0.001)	MMP-7 stronger for CT extent
Miądlikowska et al. [15]	Correlation	HRCT extent r = 0.35 (p = 0.02); higher in IPAF/CTD-ILD vs. CTD without ILD (p = 0.0005)	No independent 1-year prognostic value
Rai et al. [21]	Prognostic	Baseline SP-D 256.1 (died) vs. 191.2 ng/mL (deteriorated); serial change predicted mortality	Comparable to KL-6 for mortality
Chang et al. [16]	Prognostic	HR = 1.003 (95% CI = 1.001–1.006; p = 0.008) for RA-ILD PPF progression	Serial KL-6 change a stronger predictor
Wakamatsu et al. [19]	Prognostic	Not independently predictive on multivariate analysis	KL-6 and SP-A outperformed SP-D
Majewski et al. [23]	Prognostic	No consistent trend on antifibrotic therapy; high inter-patient variability	Uninformative for treatment monitoring

Discussion

Principal Findings

This systematic review provides a comprehensive critical appraisal of the most recent evidence on the diagnostic and prognostic value of serum SP-D in ILD, drawing on 10 original studies published between 2021 and 2025, collectively enrolling more than 4,100 participants across a broad range of ILD phenotypes, clinical settings, and geographical regions. The central finding is that serum SP-D is a biologically meaningful and clinically relevant biomarker of alveolar epithelial injury in ILD: it is elevated in the majority of ILD populations relative to controls, correlates with physiological and radiological disease severity, and carries an independent prognostic signal in specific clinical contexts. Importantly, SP-D indexes the acuity and extent of alveolar epithelial injury rather than the fibrotic phase per se, a distinction that helps explain its variable behaviour across phenotypes and treatment contexts. Its performance is not uniform across subtypes, it is consistently outperformed by KL-6 as a standalone marker in most diagnostic and prognostic scenarios, and its clinical utility is maximised within multi-biomarker strategies rather than as a solitary index test.

Diagnostic Value of Serum Surfactant Protein D

The diagnostic accuracy of serum SP-D observed across the included studies, with AUC values spanning 0.65 to 0.89, is broadly consistent with, and in some instances superior to, the performance reported in earlier foundational literature. The seminal comparative study by Ohnishi et al., which remains the reference source for the clinically applied 110 ng/mL threshold, established serum SP-D as a useful marker for ILD detection while reporting that it was less sensitive than KL-6 at comparable specificity [24]. The highest AUC reported in the present review, 0.89 by Maharjan et al. [17], was obtained in a United States CTD-ILD-enriched cohort and likely reflects the aetiological composition of that population, as CTD-ILD is characterised by profound type II pneumocyte hyperplasia and alveolar barrier disruption, the primary biological drivers of SP-D leak into the systemic circulation. These within-review observations of significant SP-D elevation across multiple populations [14,17,21] are consistent with the broader evidence base: the systematic review and meta-analysis by He et al. [25] confirmed that serum SP-D is significantly elevated in ILD relative to controls and predicts disease occurrence, progression, acute exacerbation, and mortality, validating the biological consistency of SP-D as an ILD biomarker across diverse settings. The discordant null finding from Wang et al. [20], in which SP-D failed to discriminate ILD from non-ILD controls in a Chinese cohort, is explicable by the heterogeneous ILD subtype distribution in that study and is consistent with earlier observations by Zhong et al. [26], whose meta-analysis of CTD-ILD biomarkers found SP-D to be more variable in diagnostic sensitivity than KL-6, particularly in non-fibrotic or early-stage ILD.

Serum Surfactant Protein D in Acute Exacerbation of Interstitial Lung Disease

Perhaps the most clinically impactful finding of this review is the unique behaviour of SP-D in AE-ILD, as characterised by Takeshita et al. [18]. The observation that serum SP-D, but not KL-6, correlated significantly with coagulation and fibrinolysis markers (TAT, D-dimer, PIC; r = 0.52-0.68) in AE-ILD provides direct evidence from human clinical data that SP-D may serve as a biomarker of the interface between acute alveolar epithelial catastrophe and secondary intravascular coagulopathy during AE-ILD. This is mechanistically plausible: the acute, massive release of SP-D from severely damaged type II pneumocytes during diffuse alveolar damage, the histological hallmark of AE-ILD, is likely to occur contemporaneously with activation of the extrinsic coagulation cascade mediated by tissue-factor exposure from injured endothelium and alveolar epithelium. This pathobiological linkage between alveolar epithelial injury markers and coagulopathy has been recognised in acute respiratory distress syndrome, in which SP-D levels are predictive of disease severity and 28-day mortality; the present findings extend this concept to the clinically overlapping condition of AE-ILD. A combined panel of SP-D and D-dimer may therefore have utility as a rapid, non-invasive bedside diagnostic strategy for confirming AE-ILD in patients with acute respiratory deterioration, a scenario in which HRCT confirmation is frequently delayed by haemodynamic instability or oxygen dependency. This hypothesis warrants prospective validation in dedicated AE-ILD biomarker studies.

Prognostic Value of Serum Surfactant Protein D

The prognostic findings of this review are nuanced and require careful contextualisation. The most robust prognostic evidence comes from two high-quality prospective studies: Rai et al. [21], who demonstrated that baseline SP-D and its serial change independently predicted mortality in a mixed-ILD cohort over six months, and Chang et al. [16], who identified baseline SP-D as an independent predictor of RA-ILD progression meeting internationally standardised PPF criteria (HR = 1.003; p = 0.008) within the large multicentre KORAIL registry. These findings are complementary to earlier evidence from the Japanese Myositis-Associated ILD (JAMI) cohort reported by Kaieda et al. [27], who evaluated SP-D as a mortality predictor in myositis-associated ILD and highlighted that the direction and strength of the SP-D prognostic signal may be modified by autoantibody status, differing across anti-MDA5-positive and anti-ARS-positive disease, underscoring that SP-D interpretation must be informed by the immunological context. In contrast, Wakamatsu et al. [19] found SP-D to be a non-significant prognosticator in chronic fibrosing interstitial pneumonia, and Majewski et al. [23] found SP-D uninformative for treatment-response monitoring in IPF patients on antifibrotic therapy. Taken together, these findings suggest that antifibrotic agents may decouple serum SP-D from disease activity by modulating epithelial-mesenchymal signalling and reducing acute alveolar cell turnover, thereby attenuating the biological signal that SP-D is designed to capture.

Comparative Performance Versus Krebs von den Lungen-6

The consistent superiority of KL-6 over SP-D as a standalone ILD biomarker, observed across diagnostic [17,20], functional-correlation [22], and prognostic [19, 23] domains, merits mechanistic consideration. KL-6 is a direct product of hyperplastic and regenerating type II pneumocytes and bronchiolar epithelial cells, providing a highly specific and quantitatively proportionate measure of fibroproliferative alveolar epithelial activity. SP-D, by contrast, reflects the functional state and integrity of the innate surfactant system, a property that may vary non-proportionately with fibrotic burden depending on the balance between type II cell hyperplasia, surfactant dysregulation, and inflammatory attenuation of SP-D expression. At the assay level, KL-6 benefits from a standardised latex immunoturbidimetric platform with internationally harmonised calibrators validated across multicentre studies in Japan, Europe, and North America, whereas SP-D ELISA measurements retain manufacturer-specific calibration that has not been formally harmonised, generating between-study systematic variance that inflates apparent performance heterogeneity. Despite this, SP-D captures biological information that is not entirely collinear with KL-6 and is therefore not wholly redundant in a clinical biomarker strategy: baseline SP-D predicted RA-ILD progression independently of KL-6 in multivariable regression [16], contributed complementary diagnostic value in population-level screening [14], and conveyed unique prognostic information in AE-ILD [18].

Clinical implications

From a clinical translation standpoint, the aggregate findings of this review suggest the following positioning for serum SP-D in the ILD pathway. In the diagnostic domain, SP-D at the 110 ng/mL threshold provides meaningful sensitivity and specificity for ILD detection but is insufficient as a sole diagnostic test; its greatest diagnostic impact is realised in combination with KL-6 and, in the context of population-level fibrosis screening, with AI-assisted radiographic tools [14,17]. In the prognostic domain, SP-D offers independent predictive value for disease progression in RA-ILD [16] and for mortality in mixed ILD [21], but contributes minimally to prognostication in antifibrotic-treated IPF or short-term monitoring of IPAF. In the acute clinical setting, SP-D’s correlation with coagulation markers during AE-ILD positions it as a potentially valuable component of a multi-marker AE-ILD diagnostic and severity panel [18]. Clinicians and researchers should therefore adopt a context-specific approach to SP-D measurement, recognising that its clinical value is maximised within multi-biomarker strategies informed by ILD subtype, acuity of presentation, and treatment context. From an implementation and health-economic perspective, serum SP-D is an inexpensive, widely deployable blood test that, where a validated assay is available, could plausibly reduce reliance on serial HRCT for monitoring and support earlier triage to specialist assessment; however, no included study formally evaluated the cost-effectiveness of SP-D-based testing, and the incremental cost per correctly identified or correctly stratified patient, particularly when SP-D is added to an existing KL-6 pathway, remains undefined. Formal cost-effectiveness and implementation studies are therefore required before SP-D can be recommended for routine adoption, and the mechanistic interpretations offered above (for example, the link between SP-D and coagulation activation in acute exacerbation) should be regarded as biologically plausible hypotheses generated by the included observational data rather than as established causal pathways.

Limitations

This systematic review has several limitations that must be acknowledged in interpreting its findings. The substantial heterogeneity in study populations, ILD subtypes, SP-D assay platforms, and outcome measures across included studies precluded quantitative meta-analysis, limiting the synthesis to a narrative approach and preventing the generation of pooled diagnostic-accuracy estimates or summary effect sizes. The majority of included studies had small to moderate sample sizes, with four studies enrolling fewer than 50 participants, which reduces the precision and generalisability of individual study estimates. Geographical concentration of evidence, with the majority of studies originating from East Asia and Europe, may limit generalisability to other ethnic populations, particularly given known ethnic variation in SP-D reference ranges and the predominance of the Yamasa Corporation ELISA in Asian studies. The absence of an internationally harmonised SP-D assay standard, analogous to the calibrated KL-6 latex immunoturbidimetric platform, introduces systematic between-study measurement heterogeneity that is not adjustable by any analytical approach. Four retrospective single-centre studies are additionally subject to selection bias and to limits on causal interpretation of observed associations. The review was restricted to studies published between 2021 and 2025 to ensure contemporaneous relevance; as a direct consequence, several methodologically important foundational studies that provide the mechanistic, threshold-defining, and comparative context drawn upon in the Discussion (for example, the early work cited as references [24], [26], and [27]) were deliberately outside the scope of the formal synthesis, and the conclusions of this review should be interpreted with that boundary in mind. Finally, the review protocol was not prospectively registered in PROSPERO or an equivalent registry, which we acknowledge as a limitation; the review was nonetheless conducted and reported in accordance with the PRISMA 2020 statement, with pre-specified objectives, eligibility criteria, and synthesis methods. Several further limitations warrant explicit acknowledgement. The risk of publication and small-study bias cannot be excluded: studies reporting positive or statistically significant associations for a candidate biomarker are more likely to be published, and because no quantitative synthesis was undertaken, this risk could not be formally interrogated using funnel-plot asymmetry or regression-based tests; the direction of any such bias would tend to inflate the apparent diagnostic and prognostic performance of SP-D. Relatedly, a formal GRADE assessment of the certainty of evidence was not performed, as the narrative design and the absence of pooled estimates for comparable outcomes preclude the outcome-level rating that GRADE requires; the overall certainty of the evidence base should therefore be regarded as low to moderate and the findings as hypothesis-strengthening rather than definitive. Diagnostic accuracy varied substantially across studies (AUC = 0.65-0.89), and SP-D cut-off values were inconsistently reported or, in several studies, not reported at all, which limits the derivation of a clinically actionable diagnostic threshold and further justifies the decision not to pool diagnostic estimates. The exclusion of case reports and small case series, although adopted to improve methodological comparability, may have omitted informative data on rarer ILD subtypes for which only small studies exist, so the present synthesis is most applicable to the more common fibrotic ILD phenotypes. The included evidence also originated from a limited number of countries, compounding the geographic concentration noted above and constraining generalisability to under-represented regions and health systems.

## Conclusions

This systematic review provides the most comprehensive contemporary synthesis of the evidence on serum SP-D as a diagnostic and prognostic biomarker in ILD. The accumulated evidence demonstrates that serum SP-D is consistently elevated in ILD relative to non-ILD controls and correlates with pulmonary functional impairment and radiological disease extent. Evidence for its prognostic value is more variable: SP-D carried independent prognostic information for progression or mortality in some ILD phenotypes (notably RA-ILD and mixed ILD populations) but added little in antifibrotic-treated IPF or short-term IPAF monitoring, and these prognostic signals derive largely from individual observational cohorts rather than from consistent, replicated findings across subtypes. Its diagnostic performance, while clinically meaningful, is generally inferior to KL-6 as a standalone marker, but SP-D offers non-redundant complementary value in multi-biomarker ILD strategies, particularly in AE-ILD, where its correlation with coagulation markers captures a distinct pathobiological dimension, and in population-level fibrosis screening, where its combination with KL-6 and AI imaging improves early fibrosis detection. These conclusions should be read in light of the heterogeneity in study populations, assay methods, ILD subtypes, and outcome definitions across the evidence base, and the recommendation to incorporate SP-D into multi-biomarker panels should be regarded as provisional pending prospective validation. Standardisation of SP-D assay platforms and cut-off thresholds, prospective validation of multi-biomarker panels incorporating SP-D across diverse ethnic populations and ILD subtypes, and investigation of SP-D as a pharmacodynamic monitoring biomarker during antifibrotic and immunosuppressive therapy represent the most critical priorities for future research. Until such evidence accrues, including studies using standardised assays and clinically validated cut-off values, serum SP-D is best employed as a complementary element of a multi-marker ILD assessment strategy, interpreted within the specific clinical context of ILD subtype, disease acuity, and treatment status, rather than as a standalone diagnostic or prognostic index, and its routine clinical implementation cannot yet be recommended.
